# New insights empowered by single-cell sequencing: From neural crest to enteric nervous system

**DOI:** 10.1016/j.csbj.2022.05.025

**Published:** 2022-05-18

**Authors:** Zhixin Li, Elly Sau-Wai Ngan

**Affiliations:** Department of Surgery, Li Ka Shing Faculty of Medicine, The University of Hong Kong, Pokfulam, Hong Kong

**Keywords:** Single-cell sequencing, Neural crest, Enteric nervous system, Multimodal, ENS, Enteric nervous system, scRNA-seq, Single-cell RNA sequencing, NC, Neural crest, ENCCs, Enteric neural crest cells

## Abstract

With the rapid development of single-cell sequencing technologies, it has become a powerful strategy for the discovery of rare cells and delineating the molecular basis underlying various biological processes. Use of single-cell multimodal sequencing to explore the chromatin accessibility, gene expression and spatial transcriptome has propelled us to success in untangling the unknowns in the enteric nervous system (ENS) and provided unprecedented resources for building new diagnostic framework for enteric neuropathies. Here, we summarize the recent findings of single-cell multimodal sequencing, especially focusing on the most commonly used single-cell RNA sequencing (scRNA-seq) on ENS cells, ranged from the progenitors, neural crest (NC) cells, to the mature ENS circuit, in both human and mouse. These studies have highlighted the heterogeneity of ENS cells at various developmental stages and discovered numerous novel cell types. We will also discuss various computational methods that were used to reconstruct the differentiation trajectories of the developing ENS and to elucidate the cell fate decisions. Profiling disease mechanisms and cellular drug responses with single-cell multimodal omics techniques likely leads to a paradigm shift in the field of biomedical research. Further improvements in the high-resolution sequencing platforms and integrative computational tools will greatly hasten their applications in both the basic and translational medicine.

## 1 Introduction

Enteric nervous system (ENS) comprises functional neural circuits containing many different types of neurons and glia that are organised into two interconnected plexuses of ganglia: the myenteric and submucosal plexuses in the gut. It coordinates intestinal motility, digestion, local immunity and inflammation [1]. The majority of enteric neurons and glia are derived from the vagal neural crest (NC) cells, albeit some are derived from the sacral NC cells. In mice, when the NC cells reach embryonic foregut at embryonic day (E) 9 (corresponding to 4 weeks gestation in human), they are termed as enteric neural crest cells (ENCCs) given their specialized location and functions in the gut. ENCCs migrate in a rostral-to-caudal direction to sequentially colonize the foregut, midgut and the hindgut and the colonization is completed by E15 (corresponding to 7 weeks gestation in humans). Sacral NC cells start colonizing the gut at later stage and contribute to only a small fraction of enteric neurons and glia in the distal midgut and hindgut [2]. While the ENCCs are migrating to colonize the entire gut, they undergo extensive proliferation and differentiation in order to give rise to a full diversity of neurons and glia for the assembly of functional ENS circuit. Other than ENCCs, Schwann-cell like precursor cells were identified more recently and they contribute to a significant portion of enteric neurons in both the anterior part (esophagal and stomach) [3] and more distal regions [4] of the gut.

A broad range of human intestinal diseases are associated with ENS dysfunction. Hirschsprung disease is the most common congenital enteric neuropathy, characterized by the incomplete colonization of the bowel by ENCCs, resulting in the colonic aganglionosis [5]. Abnormality of ENS also causes other enteric motility disorders, such as immune-mediated loss of neuronal subtypes in esophageal achalasia and Chagas disease, degenerative neuropathies in some cases of chronic intestinal pseudo-obstruction and gastroparesis, in which enteric ganglion cells are present and absence of any occlusive lesion [6]. These ENS disorders affect both paediatric and adult patients.

Among various high-resolution single-cell sequencing platforms, the single-cell RNA sequencing (scRNA-seq) is the best established technology as of today and has been widely used to study various system organs. It gives a global view of the cell heterogeneity and allows reconstruction of differentiation trajectories underlying the early embryonic development of the organisms [7], [8]. Building the cellular roadmaps of every cell type in human or other model species will help disentangle the complexity of cells in diverse tissues and conditions [9], [10]. The single-cell multimodal omics analyses integrating the genetics, epigenetics, transcriptomics, proteomics and the spatial and functional characteristics of each cell at a time further allow a holistic understanding of biological networks in heterogeneous tissues [11]. In this review, we will summarize the uses of single-cell RNA sequencing technologies, albeit few studies also included single-cell multimodal omics analyses, on understanding the ENS cells at various developmental and adult stages in human and mouse, and highlight the major discoveries of these studies (Table 1). The challenges and future directions of the field will also be discussed.

**Table 1:** Key profiles of the 21 single-cell sequencing studies of ENS.

	Species	Stage	Cell source	Cell sorting	Platform	Cell number	Year	Reference	Website
Embryonic/Fetal	Mouse	E6.5–8.5	Whole embryo	NA	10X Genomics	116,312*	2019	Pijuan-Sala et al. [13]	link
	Mouse	E8.5–10.5	Whole embryo	*Wnt1^Cre^/R26R^Tomato^*	SMART-seq2	6124*	2019	Soldatov et al. [15]	link
	Mouse	E9.5–13.5	Whole embryo	NA	sci-RNA-seq3	2 million*	2019	Cao et al. [16]	link
	Mouse	E12.5–13.0	Small intestine	*Sox10::CreER^T2^;Rosa26tdTomato* (SER93|tdT)	SMART-seq	120	2017	Lasrado et al. [19]	NA
	Mouse	E13.5	Whole bowel	*Wnt1-cre;YPF*	10X Genomics	7671	2019	Lau et al. [20]	NA
				*Tg(GBS-GFP)*		2017			
	Human	20, 40 days	hPSC-derived NC derivatives	HNK-1 and p75^NTR^	SMART-seq2	2726			
	Mouse	E13.5	Whole bowel	Wnt1-Cre;Rosa26^YPF^	10X Genomics	7671	2021	Lai et al. [17]	NA
				*Wnt1-Cre;Kif7^f/f^;Rosa26^YPF^*	10X Genomics	15,522			
	Mouse	E14.0	Whole embryo	NA	sci-Space	121,909*	2021	Srivatsan et al. [18]	NA
	Mouse	E15.5–18.5	Small intestine	*Wnt1-Cre;R26R-Tomato*	10X Genomics	5993	2021	Morarach et al. [31]	NA
		P21	Small intestine Myenteric plexus	*Baf53b-Cre;R26R-Tomato*		9141			
	Mouse	E17.5	Whole bowel	*ChAT-EGFP-L10A+* *Nos1-CreERT2^Cre/wt^;R26RTdTomato+*	10X Genomics	1005	2021	Wright et al. [32]	NA
		7 weeks	Distal colon myenteric plexus	*Wnt1-cre^cre/wt^; R26R-H2B-mCherry^ch/wt^*		1549			
	Human	Adult	Colon myenteric plexus and surrounding cells	Hoechst+		20,167*			
	Human	8–22 PCW	Colon	NA	10X Genomics (scRNA-seq)	76,592*	2021	Fawkner-Corbett et al. [36]	link
		12, 19 PCW Adult			10X Genomics Visium Spatial	NA			
	Human	6–17 PCW	Small and large intestine	NA	10X Genomics	62,854*	2020	Elmentaite et al. [39], [40]	link
						428,000*	2021		
	Human	7–21 PCW	Small intestine	NA	10X Genomics	24,783*	2021	Holloway et al. [38]	NA
	Human	10–18 PCW	15 organs (Intestine)	NA	sci-RNA-seq3	4 million*	2020	Cao et al. [34]	link
					sci-ATAC-seq3	800,000*	2020	Domcke et al. [35]	

Postnatal	Mouse	P14	Small intestine LMMP	*Plp1::GFP*	10X Genomics (scRNA-seq)	13,936	bioRxiv 2021	Guyer et al. [43]	NA
		P16-18			10X Genomics (snATAC-seq)	10,748			
	Mouse	P21	Small intestine Myenteric plexus	*Wnt1-Cre;R26Tomato*	10X Genomics	492,949* (ENS: 11,640)	2018	Zeisel et al. [21]	link

Adult	Mouse	6–7.5 weeks	Myenteric plexus	*Phox2b-CFP*	10X Genomics (snRNA-seq)	25,208	2021	May-Zhang et al. [45]	NA
	Mouse	10–12 weeks	Colonic extrinsic sensory neurons	Retrograde tracer	SMART-seq2	399	2019	Hockley et al. [48]	NA
	Mouse	11–104 weeks	Colon	Wnt1-Cre Sox10-Cre Uchl1-H2B mCherry	RAISIN RNA-seq	5696	2020	Drokhlyansky et al. [44]	NA
			Ileum and colon	NA	MIRACL-seq	3558			
	Human	35–90 years old	Colon *muscularis propria*	NA		7499			
	Human	7–26 PCW	Intestine	NA	Microwell-seq	702,968*	2020	Han et al. [50]	link
		21–83 years old							

*indicates the single-cell sequencing was not specifically designed for ENS study, but it still contained valuable neural crests or ENS cells for further study. LMMP, longitudinal muscle-myenteric plexus. E, embryonic day; P, postnatal day; PCW, post-conception weeks. NA, not applicable.

## 2 Insights into ENS as revealed by single-cell sequencing

In the recent 5 years, tremendous efforts have been made to generate the single-cell atlas of ENS at developmental and adult stages in human and mouse. Various single-cell sequencing tools, stage/cell-specific reporter mouse lines, isolation methods for cell enrichment/isolation and analytic strategies have been applied for the discovery of rare cells and delineating the molecular basis of neuron specification and diversification. In this part, we summarize the main insights and new knowledge acquired from the single-cell sequencing of ENS in different developmental stages.

### 2.1 ENS during prenatal/fetal development

Mouse is commonly used as an experimental model to study human development. Unlike human, mice develop quickly with just 19–21 days between fertilization and birth. During the transition from gastrulation to early organogenesis between E6.5 and E8.5, NC cells delaminate from neural tube and give rise to a diverse array of cell lineages, including the progenitors of ENS [12].

In 2019, Pujuan-Sala et al. profiled 116,312 single cells from mouse embryos from E6.5 to E8.5 to construct a molecular map of cellular differentiation from pluripotency towards all major embryonic lineages [13] (interactive website: https://marionilab.cruk.cam.ac.uk/MouseGastrulation2018/). They identified a population of NC cells which uniquely expressed Sox10. Time-series scRNA-seq analysis showed that the NC population emerges at E8.25 and develops quickly at E8.5. These observations are in line with the previous descriptions that vagal NC lineage is first observed at E8.5 in mouse embryos [14]. Therefore, this dataset would be useful for elucidating the molecular events underlying the development of vagal NC cells during gastrulation and early organogenesis.

In the same year, Soldatov et al. used single-cell and spatial transcriptomics to identify the cell fate decisions in murine NC between E8.5 to E10.5 [15] (interactive website: http://pklab.med.harvard.edu/ruslan/neural.crest.html). In this study, the authors conducted an intensive hypothesis-driven bioinformatic analysis on scRNA-seq data and provided valuable insights into NC fate commitment. Through combining clustering, trajectory and *in situ* sequencing analyses, they reconstructed and validated a dynamic differentiation process of NC, began with the pre-epithelial mesenchymal transition (EMT) state and NC delamination, followed by the divergent differentiation of migrating progenitors toward autonomic and sensory neuronal and mesenchymal lineages. Applying principal graph approach for branch analysis, they identified two steps of bifurcation during NC differentiation, the first one separates sensory lineage from the common progenitors of autonomic and mesenchymal branches, and subsequently the second fate split separates autonomic neuronal fate from mesenchymal lineage. Gene modules associated with each branch were further identified, in which *Neurog2* was shown to possess different functions at early- and late- pseudotime. For the bifurcation process of sensory and autonomic branch, they identified both coactivation and competing gene modules. Synchronized with the continuous process of cell state transition, the gradual expression shifts were also observed between competing fate-specific modules. Therefore, they concluded that the NC fate decision contains three successive phases: initial coactivation, gradual biasing, and final commitment. Similar differentiation pattern was also identified in vagal and tail (sacral) NC cells in this study.

The high resolution transcriptomic data of mouse ENS cells during organogenesis were also described in another study published in 2019. Cao et al. reported the transcriptomes of around 2 million cells derived from 61 embryos staged between E9.5 and E13.5 to investigate the transcriptional dynamics of mouse organogenesis at single-cell resolution [16] (interactive website: http://atlas.gs.washington.edu/mouse-rna). In the trajectory analysis, they identified two major trajectories of NC-derived glia and neuron for the peripheral nervous system (PNS), in which four sub-trajectories were further classified as enteric glia and neurons. Since this study did not report any specific analysis on the ENS cells, our group re-analysed this dataset utilizing the latest integration method developed for scRNA-seq data, and particularly focused on cells on the ENS lineages. ENS cells from multiple developmental stages were classified into three main populations (BP: bipotent progenitor; NP: neuronal progenitor; GP: glial progenitor) [17]. The proportions of these three cell populations dynamically change along the developmental stages. Intriguingly, benefit from the integrated analysis, we found *Ezh2* is up-regulated in ENCCs on the glial lineage but down-regulated in the cells on the neuronal lineage, where Ezh2 acts as a transcription activator and repressor for gliogenic and neurogenic lineage differentiation of ENCCs, respectively. It is noteworthy that the cell proportions inferred by scRNAseq should be carefully validated by immunohistochemistry with specific markers. In many cases, inefficient isolation of single neurons from tissue specimens may make it difficult to quantify the relative population sizes accurately from single-cell sequencing data.

The mouse organogenesis cell atlas dataset established by Cao et al. was also used by another group for an integrative analysis with the spatial transcriptome data. Srivatsan et al. applied spatial single-nucleus RNA-seq (snRNA-seq) to developing mouse embryos at E14.0 to depict the anatomically expression pattern of cells [18]. Sequenced nuclei were localized with spatial positions across each sagittal sections. They found that different types of cells exhibit a substantial difference in their spatial patterning, and the pseudotime of differentiating neurons can be correlated to their migratory patterns. This technology allows the visualization of the expression of any gene in the sections using an “digital *in situ*” approach. Followed by the clustering analysis, the sci-Space is able to resolve spatial gene expression by cell type. These datasets are valuable for further in-depth study of the ENS development.

scRNA-seq data on ENS cells at later stage were published in 2017, it is also the first study characterizing the isolated ENS cells with high-resolution RNA-seq. Lasrado et al. profiled 120 single-cell transcriptomes of ENS cells from E12.5 to E13.0 *Sox10::CreERT2;Rosa26tdTomato* (*SER93|tdT*) mouse embryonic guts to investigate the transcriptional signatures of the developing ENS cells and the molecular regulators directing the neuronal and glial lineage differentiation. Through the PCA analysis, they identified an undifferentiated ENS progenitors (BP) which can continuously differentiate along either glial (*Erbb3, Sox10, Fabp7, and Plp1*) or neuronal (*Tubb3, Elavl4, Ret, and Phox2b*) lineages [19]. Based on the expression of the neuronal and glial markers, they further subdivided the undifferentiated progenitors cells to early transient stage of the neurogenic lineage (co-expressed the progenitor/neurogenic and progenitor/gliogenic modules) and uncommitted progenitors (expressed the progenitor/gliogenic markers but were generally negative for *Tubb3* and *Elavl4*). Through the gene correlation analysis with *Tubb3* and *Erbb3*, they identified key genes associated with neurogenesis and gliogenesis. This study first provided a molecular view of ENCC dynamics and identified *Ret* as a regulator of neurogenic commitment. However, only 120 cells were sequenced in this study, the small sample size limits the differentially expression analysis and hampers the identification of the full heterogeneity (rare subtypes) of the ENS cells.

More cells (7,671) at the same stage have been sequenced using 10× Genomics and published in 2019, where ENS cells were isolated from the E13.5 *Wnt1-Cre; YFP* mouse guts [20]. Consistent with the above study [19], four major groups of ENS cells were identified in mouse embryonic guts at E13.5, including BP, NP (*Tubb3^+^/Elavl4^+^/Cartpt^−^/Prph^−^*), GP (*Sox10^+^/Fabp7^+^*) and a population of non-neural cells, namely the enteric mesothelial fibroblasts [21]. This dataset was then used to preform comparative studies to delineate the implications of Hedgehog (Hh) signaling in directing cell progression and/or lineage commitment.

Hh signalling has been implicated in various stages of ENS development and its aberrant activation alters the neuron-to-glia ratio in mouse ENS and that is associated with HSCR pathogenesis [17], [22], [23], [24], [25], [26]. Thus, high-resolution scRNA-seq analyses were performed using ENS cells isolated from various mouse models to further delineate its role in ENS development. In the first study, a mouse reporter line *Tg*(*GBS-GFP*) was used, in which GFP expression is driven by GLI transcription factors that bind to the eight concatemerized GLI-binding sequence (GBS) [27], to monitor the *in vivo* GLI activity (a readout of Hh signaling) in ENS cells. E13.5 represents a key window for early development of ENS in mice, where two distinct neuronal and glial lineage differentiation trajectories are established [19] and many genes implicated in early ENS development and the associated congenital diseases can be studied in this window. Therefore, the single-cell transcriptomes of GFP^+^ (activated Hh signaling) and GFP^−^ (inactive Hh signaling) ENS cells from E13.5 *Tg(GBS-GFP)* guts were profiled. Using an integrative approach connecting clustering with differentially expressed genes (DEG) analysis, the key direct target genes of Hh pathway were found to be predominantly up-regulated in the enteric BP cells, suggesting a potential involvement of Hh signaling in cell state transition of ENS development, particularly, on directing the enteric BP to transit from the bipotent state to the neuronal fate [20].

A related study was published 2 years later, where another key molecule implicated in Hh signalling transduction, Kinesin family member 7 (Kif7) was studied using mouse mutants with conditional deletion of *Kif7* in NC. The mutant mice exhibited growth retardation and gut dysmotility due to a reduction in the percentage of nNOS^+^ inhibitory neurons in the postnatal ENS. Ablation of *Kif7* affected the early stage of ENS development by reducing the numbers of NP cells and disrupting ENCC migration. Through analysing the single-cell transcriptomes of control and *Kif7* mutant ENCCs at E13.5, Kif7 was found to be implicated in initiating the neuronal lineage differentiation through regulating Gli and Ezh2 [17].

scRNA-seq data generated from the mouse studies have also been used for establishing human pluripotent stem cell (hPSC)-based model of ENS. hPSC-based model have been widely used for disease modelling. Various directed differentiation protocols have been established for the derivation of posterior NC cells from hPSC [28], [29]. To further validate this system, single cell transcriptomes of NC cells derived from hPSC and their NP derivatives after 20 and 40 days of neuronal differentiation were compared to the mouse ENS cells. PCA and correlation analysis revealed that hPSC-derived NC give rise to NPs resembling the developing ENS in E13.5 mouse embryonic gut, they shared a similar NC-to-NP transition trajectory and the transcription network [20]. This study has also demonstrated that Hh can improve the neuronal differentiation of NC cells derived from hPSCs by priming them at a higher cell state during the NC-to-neuron transition as revealed by scRNA-seq analysis.

In mouse, most of subtypes of the enteric neurons emerge during E15.5 and E18.5 and more than 90% of them are either nitrergic (nitric oxide–producing, Nos1^+^) or cholinergic (acetylcholineproducing, ChAT^+^) neurons [30]. In 2021, Morarach et al. performed scRNA sequencing to depict the enteric neuronal diversification within the myenteric plexus of the mouse small intestine from E15.5 to 18.5 [31]. They classified the ENS cells into four main stages, including stem cell maintenance, neurogenesis, neuron differentiation and glia differentiation. The Uniform Manifold Approximation and Projection (UMAP) revealed the enteric neurons are formed through two trajectories which give rise to different types of neurons. Therefore, they proposed a novel model of enteric neuron specification with a hypothesis that all enteric neurons are generated through a binary differentiation. Most nitrergic (*Nos1*^+^) neurons were found at early stage of branch A and three clusters of cholinergic neurons were formed through a nitrergic-to-cholinergic (*Slc18a2^high^*) switch process at later stage of branch A, while branch B mainly comprised cholinergic neurons (*ChAT*^+^). Subsequent analysis further identified *Pbx3* as an key transcription factor which regulates the transition of post-mitotic inhibitory to excitatory enteric neurons. In sum, this study demonstrated that the segregation between nitrergic and cholinergic neurons is established as early as E15.5. In particular, at this stage, a proportion of *Nos1*^+^ neurons is actually transiting to *NOS1*^−^,*ChAT^+^* neurons, while many neurons have achieved terminal differentiation.

In addition to ENS in small intestine, sequencing ENS cells in the distal colon would also be informative. In 2021, Wright et al. performed another sequencing analysis and particularly focused on the ENS cells on ChAT-lineage and Nos1-lineage in E17.5 distal colon. They aimed to identify genes that are specifically expressed in these two subtypes of neurons and the corresponding transcription factors regulating the lineage specification [32]. Other than the epithelial and muscle cells, they identified 8 clusters of neurons including three clusters representing the less mature neurons and 5 clusters of mature neurons (4 *ChAT*^+^ clusters and 4 *Nos1*^+^ clusters). Through differential analysis of gene expression, they identified a set of key genes including neurotransmitters, receptors, ion channel subunits, and signaling molecules differentially expressed between neuron subgroups, including *Gfra1* and *Gfra2.* A substantial overlap was found between E17.5 and adult subgroup on the expression of regulatory genes such as *Casz1*, *Bnc2*, *Etv1*, *Pbx3*, *Tbx2*, *Tbx3*, and *Rbfox1*. Among those, *Tbx3* was identified as a transcription factor associated with the formation of *Nos1+* neurons and loss of *Tbx3* in ENS led to reduction in Nos1^+^ neurons in small intestine.

In human, at post-conceptual weeks (PCW) 7–8, corresponding to E15 in mouse, colonization of the gastrointestinal (GI) tract by ENCCs is completed, while the formations of myenteric ganglia is still ongoing [33]. Cao et al. and Domcke et al. generated single-cell atlases of both gene expression and chromatin accessibility from 15 human organs during mid-gestation (10–18 PCW) [34,35] (interactive website: https://descartes.brotmanbaty.org/). They integrated these human fetal data with a mouse organogenesis cell atlas at E9.5 and E13.5 [16] and identified two trajectories of NC-derived PNS- glia and neurons which contained the enteric neuron and glia clusters as well. Furthermore, they performed multimodal integration of single-cell data to map the single-cell chromatin accessibility data to the single-cell transcriptome data and identified distinct chromatin landscapes for enteric neuron and glia clusters. These integrated data allow us to identify cell type-specific transcription factors and the regulatory networks of their target genes. More in-depth analyses are required to define the transcriptional regulatory networks underlying ENS development using these valuable datasets.

More recently, Fawkner-Corbett et al. performed scRNA-seq and spatial transcriptomics (ST) analyses to create a single-cell spatiotemporal atlas of human intestinal development using dataset ranged from 8 to 22 PCW [36] (interactive website: https://simmonslab.shinyapps.io/FetalAtlasDataPortal/). They identified several progenitors cells including ENS progenitors and glial progenitors, and an abundance of more differentiated ENS cells including inhibitory motor neuron precursors, inhibitory motor neurons, excitatory motor neurons, interneurons, three clusters of glial cells as early as 8–10 PCW. The proportion of ENS progenitors and glial progenitors were found decreased along the development. The cell type interaction between ENS cell and non-ENS cells were predicted based on receptor-ligand pairs in which LEFTY1-ACVR2B and TPH1-HTR2B are the two key signalings active in ENS cell types, the latter is a known serotonin signalling pathway mediating appetite/motility [37]. In this study, ST was also performed to map transcriptional signatures to distinct geographical regions. Through carrying out a factor analysis, the authors successfully aligned *RET* expression at myenteric plexuses using ST data. ENS only represents a minor population in the intestinal tissue and only few ENS cells were captured for ST analysis in this study. Nevertheless, ST would provide insights into the potential cross-talks between ENS with their neighboring cells, and that would be complementary to other scRNA-seq datasets generated from the enriched ENS cells, giving a better sense of the whole.

In the same year, Holloway et al. interrogated developing human intestine from 7 to 21 PCW with both transcriptional and spatial profiles and investigated the heterogeneity of developing human intestinal stem cell niche, especially for the mesenchymal cell populations [38]. Enteric neurons were consistently identified from fetal human duodenal enteroids at nine discrete development stages. However their analysis mainly focused on the mesenchymal niche populations. Further analysis is needed to explore the full heterogeneity of enteric neurons at various developmental stages in human.

Two more studies on human ENS covering various developmental stages have been published in 2020 and 2021 by another group. Elmentaite et al. have profiled 62,854 and 11,302 terminal ileal cells from prenatal and neonatal guts, respectively, to understand the gut development in human [39] (interactive website: https://www.gutcellatlas.org/). They identified ENCCs and enteric neuron clusters in embryonic and fetal stages, while glial cells were only detected in neonatal stage. A year later, the same group reported a more comprehensive analysis. 428,000 cells from the small and large intestines at fetal, neonatal and adult human guts have been profiled and used to map cell lineages in gut [40]. Based on UMAP, they identified the main ENS trajectories at different developmental stages. In early development, at 6–11 PCW, ENCCs differentiated primarily to neurons via neuroblasts, giving rise to two distinct branches: branch A (*ETV1^+^*) and branch B (*BNC2^+^*). Branch A further differentiated to inhibitory motor neurons and two subsets of neurons resembling IPANs or interneurons, while Branch B mainly gave rise to immature excitatory motor neuron (eMN) subsets. At later stage of development, branch A differentiated into NEUROD6-expressing interneurons, whereas branch B formed IPANs. It is very similar to differentiation trajectories observed in mice as described in another study [31].

### 2.2 ENS at neonatal stage

In mouse and human, ENS development persists after birth and enteric neurons continue to be born in the postnatal and mature gut [41]. Therefore, it is of a particular interest to characterize the postnatal gut using scRNA-seq. The first high-resolution RNA-seq study on neonatal ENS was published in 2018. Zeisel et al. isolated and sequenced more than 10,000 ENS cells from small intestine of *Wnt1-Cre;R26Tomato* mice on postnatal day of life 21 (P21) [21] (interactive website: http://mousebrain.org/). They identified 9 types of enteric neurons (3 *Nos*+ nitrergic and 5 *ChAT*+ cholinergic), 7 types of enteric glia cells (EGCs, named as ETG in original reference) and a population of enteric mesothelial fibroblasts. The enteric glia are very abundant which account for 91% of all enteric cells, including a group of EGC progenitors which is highly proliferative (*Top2a*+). Although enteric neurons are commonly divided into nitrergic and calretinin-expressed subtypes, their data indicated a more natural split occurs between nitrergic (*Nos1*^+^) and cholinergic (*ChAT*^+^ and *Slc5a7*^+^) neurons.

This P21 scRNA-seq dataset was subsequently used in another study, where multiple scRNA-seq datasets generated from different sequencing platforms were included in the analysis [17]. This study included ENS cells at early developmental stages ranged from E9.5 to E13.5 [16], E16.5 and P21 [21] and built an unified development trajectory of ENS development. This analysis gave an overview of cell populations along various developmental stages. In particular, it showed that glial cells emerge at around E12, increase dramatically during ENS development and become the dominant population of cells at the postnatal stage.

To follow up on their own data, Morarach et al. used another reporter mouse line (*Baf53b-Cre;R26R-Tomato*) to mark the enteric neurons in P21 guts. Single cell transcriptomes of 4,892 neurons were analyzed and identified 12 enteric neuron classes (ENCs) [31]. They performed probabilistic cell-type assignment to map these ENCs to previously functional enteric neuron types and annotated these 12 ENCs as excitatory inhibitory, motor neurons, intrinsic primary afferent neurons (IPANs), cholinergic and nitrergic interneuron, somatostatin interneuron and serotonergic interneuron. Expressions of glutamatergic and GABAergic markers were also detected in some of these ENCs. They further explored the neuronal subtype-specific features such as enteric neurotransmission, ion channels and transcription factors to confirm the identity of the ENCs. The existence of ENCs *in vivo* were further validated by immunohistochemistry in mouse small intestine. To understand how these 12 ENCs are generated, they anchored the 12 ENCs identified at P21 to the development trajectories constructed with ENS cells at E15.5 and E18.5 and successfully located these 12 ENCs in two bifurcated branches of differentiation trajectory. Among these 12 ENCs, two of them emerge mainly postnatally. Key transcription factors that control the a binary fate split during neurogenesis were also analyzed. Till now, this is the most comprehensive and in-depth study that revealed the bifurcated fate commitment and heterogeneity of enteric neurons at both embryonic and postnatal stages.

EGCs are an important population of cells in ENS, they are not only working as the supporting cells, but also playing regulatory roles and some are possessing neurogenic potential [42]. In 2021, Guyer et al. performed scRNA-seq and single nucleus assay for transposase-accessible chromatin using sequencing (snATAC-seq) on Plp1-expressing EGCs from the mouse small intestine at P14 and 16–18 to characterize the full diversity of EGCs [43]. They identified nine transcriptionally distinct subpopulations of EGCs, including cells expressing both canonical neural stem cell genes (glial neuroblast) and enteric neuronal transcriptional factors. Multimodal integrative analysis further revealed that these EGCs maintain a chromatin structure primed for neuronal fate acquisition and it would be the regulatory basis for the glial-to-neuronal transition. This is the first application of integrative scRNA-seq and snATAC-seq to enteric glial cells, which will greatly extend our understanding of the functions and regulatory roles of glial cells in ENS.

### 2.3 Adult ENS

Enteric neurons comprise less than 1% of colon cells in adult guts. They are tightly surrounded by muscle cells which makes it difficult to be isolated and enriched for single-cell sequencing. To tackle this technical challenge, Drokhlyansky et al. developed two methods to enable snRNA-seq of the ENS: ribosomes and intact single nucleus (RAISIN) RNA-seq for isolation of intact nuclei and ribosome-bound RNA and mining rare cells sequencing (MIRACL-seq) for label-free profiling of rare cell types, such as enteric neurons [44]. They profiled 2,657 neurons and 3,039 glia from adult mice (11–104 weeks) using RAISIN RNA-Seq with SMART-Seq2. The authors suggested that the enteric neurons can be divided into five groups including 5 excitatory motor neurons (PEMNs), 7 inhibitory motor neurons (PIMNs), 4 sensory neurons (PSNs), 3 interneurons (PINs) and 2 secretomotor/vasodilator neurons (PSVNs). Glia were clustered into 3 subsets based on the differentially expressed receptors and transporters. However, further functional assessments are needed to confirm the annotations of these cell groups. To capture differences along the proximal–distal axis of the colon, they divided each colon specimen into four equal segments and found ENS composition varied by their locations. Further, they identified ENS expression programs influenced by circadian phase, gender and age. Empowered by MIRACL-seq, they profiled 704,314 mouse colon nuclei and successfully identified rare ENS cell types including 1,938 neurons and 1,620 glia in the ileum and colon without using the transgenic mice. Although neuron subsets from the ileum and colon were congruent, they differed significantly in their proportions and aspects of their gene expression programs. Putative interactions between ENS and diverse types of gut cells were also addressed.

Later, Wright and colleagues published their ENS scRNA-seq data on the younger adult mice (47–52 days, ∼7 week-old). Again, to improve the yield of ENS cells for sequencing, they obtained the myenteric plexus nuclei from the cryosections of the distal colons. The 10x Genomics platform was used to generate snRNA-seq data from 1,520 mCherry^+^ neuronal and glial nuclei [32]. A total of 12 clusters were identified: 4 clusters of 4 glia (*Plp1+, Sox10+*), 4 clusters of neurons (*Elavl4*^+^), and 4 types of non-ENS cells including PDGFRa^+^ cells, interstitial cells of Cajal [ICC] and smooth muscle cells [SMCs]. They then further subdivided the neuronal cells into 7 clusters including two neuron groups expressing *Nos1*, *Vip* and *Gal*, four groups of ChAT^+^ neurons and another group of neurons expressing *Calcb*, *Grp* and *Nmu*. They also found that many neurotransmitters and their receptors are differentially expressed between neuron subgroups. Gfra1 (receptors for Gdnf) was associated with *Nos+* neurons while Gfra2 (receptors for Nrtn) was primarily in *ChAT+* neurons. In addition, they demonstrated that Gdnf and Nrtn induce distinct myenteric neurons where Gdnf acutely enhances colon contractility. Transcription and splicing factors were also used to define neuron subtypes. In this study, they also sequenced single nuclei from the enteric ganglia and the surrounding cells in the adult human colon. Among the 20,167 sequenced nuclei from 16 adult colons, expressions of neuronal genes were only detected in 48 cells. These neuronal cells could be further subdivided into two clusters based on the expressions of *NOS1/VIP/GAL*. The authors indicated that large amounts of doublets were detected, likely due to the incomplete dissociation of the tissues during sample preparation.

In the same year, May-Zhang et al. published another set of data on adult ENS. They performed both bulk RNA-seq and snRNA-seq on adult human and mouse enteric neurons isolated from different regions of the bowels to identify markers for discrete and conserved neuron subtypes in humans and mice [45]. They identified 22 distinct enteric neurons throughout the entire intestine including intrinsic primary afferent neurons (IPANs), descending interneurons, excitatory and inhibitory motor neurons in adult mouse guts. Several neuronal subtypes were found to be conserved between human and mice, such as *NMU*+ IPANs. However, the authors have also proposed that different types of IPANs may exist in mice and human based on the expression of *Klhl1* and the Dogiel Type II morphologies, additional analyses are needed to confirm these observations. Moreover, some conserved and disparate region-specific markers, gender- and age- related genes were identified from human and mouse intestines. In sum, this study offers the precious resources to improve our understanding of the heterogeneity and complexity of human and mouse ENS across multiple bowel segments.

Sensory neurons in the gut are responsible for transducing mechanical or chemical stimuli of the bowel into neural reflexes within the gut and outside the gut to the spinal cord and brain. There are two main classes of sensory neurons in the gut: the intrinsic sensory neurons that entirely located in the ENS, and extrinsic sensory neurons comprising cell bodies outside the gut wall with nerves endings in the gut [46]. The behavioural changes induced by the GI tract in response to the damages rely on the extrinsic sensory neurons through the autonomic pathways, and that subserves an essential role in providing conscious awareness of injury [47]. In 2019, Hockley et al. reported the scRNA-seq data on extrinsic sensory neurons in mouse colon and identified seven groups of colonic sensory neurons including 2 lumbosacral and 5 thoracolumbar subtypes [48]. Based on the gene expression patterns, they further annotated them into three peptidergic clusters, three neuro-filament clusters and one non-peptidergic cluster. The abundance of the subtypes was different between lumbar splanchnic and pelvic, suggesting different stimuli transduced by these sensory neurons. They also found that different sensory neurons have selective expression of serotonergic signalling pathway genes. This study offers critical information to guide the drug screening studies for bowel disorders, such as irritable bowel syndrome (IBS).

Baghdadi and colleagues have recently demonstrated that some EGCs are residing adjacent to intestinal stem cells and work as a stem cell niche to support the regeneration of intestinal epithelium in adult gut [49]. In this study, they analyzed the mouse and human intestinal mucosa transcriptomes at the single-cell level, and discovered 3 clusters of EGCs. These 3 clusters of EGCs are functionally redundant and crucial for intestinal homeostasis. Using a mouse model of chronic inflammatory bowel disease, they further demonstrated that a specific subset of GFAP+ (glial fibrillary acidic protein) EGCs are required for intestinal epithelium regeneration upon injury.

Some additional ENS scRNA-seq data can also be found in another large-scale study, where 702,968 single cells on 60 human fetal and adult tissues been profiled to dissect cellular heterogeneity in complex system [50] (interactive website: http://bis.zju.edu.cn/HCL/). They sequenced intestine cells at fetal stage and included multiple anatomical regions including ileum, jejunum, duodenum, rectum, ascending colon, transverse colon and sigmoid colon. They identified 18 cell clusters of cells in the colon including *CCL2*+ enteric nerve cells. In the same year, He et al. reported another set of data covering the transcriptomes of 84,363 single cells derived from 15 tissue organs including small intestine and rectum from one adult donor to reveal the inter- and intra-organ tissue heterogeneity [51]. However, the authors did not separate ENS cell from other cells, this make it difficult to be used for studying ENS.

## 3 Single-cell technologies applied to ENS studies

Methods for scRNA-seq can be divided to two categories based on their throughput capacity: the low-throughput plate-based method where cells are sorted into multi-well plates; and the high-throughput droplet-based method where cells are in suspension and each cell is compartmentalized into a droplet with barcoded beads that can be further recognized by computational algorithm. Another supremely powerful sequencing platform is using an ultra-high-throughput combinatorial indexing approach where single cells are barcoded multiple times, and that allows the large-scale sequencing of complex tissues, and it is good for sequencing cells in the whole embryo or organ in high resolution [52].

Based on the origin of the genetic material, the techniques can be divided into single-cell or single-nucleus based RNA-seq. Several tissues, like brain, skeletal muscle and adipose, are difficult to be dissociated into a single-cell suspension and are usually stored as frozen sample, which make them often not available for single-cell based sequencing [53]. To tackle this technical challenge, single-nucleus RNA-seq was established to minimize the alteration of gene expression during sample processing [54]. Comparison studies also showed that snRNA-seq possess a high sensitivity and make the data suitable for classification of cell types [55], [56].

Among the 21 single-cell sequencing studies that we have discussed above, half of the them adopted the droplet-based 10 × Genomics platform, considering its merit of high-throughput, labour saving and relatively low cost (Table 2). However, sometimes plated-based scRNA-seq is a better choice when the cell samples are precious and limited, or it requires a high read coverage to detect low-expression genes and splicing events among cells [57]. Intending to build cell atlas of organs or whole embryo, the ultra-high-throughput approach, like single-cell combinatorial indexing RNA sequencing (sci-RNA-seq), is needed [16], [34], [35]. To complement with the single-cell transcriptomic data, methods for profiling chromatin accessibility and spatial transcriptome at single-cell level have also been developed and the single-cell multi-omics technologies will further improve our ability to interrogate the molecular networks in ENS [18], [36].

**Table 2:** Summary of three representative single-cell sequencing technologies applied in ENS study.

scRNA-seq method	Category	Throughput	Coverage	Detected gene number	Cost
SMART-seq	Plated-based	Low (up to 5000 cells)	High	5000–10,000 per cell	High
10X Genomics	Droplet-based	High (up to 5 million cells)	Medium	3000–6000 per cell	Medium
sci-RNA-seq	Droplet-based	Ultra-high (up to 50 million cells)	Low	100–1000 per cell	Low

## 4 Future perspectives

Since the first single-cell RNA sequencing of ENS cells was published in 2017, many more single-cell sequencings were conducted over the last 5 years, covering nearly all periods of ENS development from NC cells to full functional ENS circuit in human and mouse. Cell heterogeneity was discovered and numerous rare cell types were identified at various developmental stages of ENS. Crucial fate decisions at early development of ENS was modelled by computational methods. However, the parameters used for clustering are still arbitrary, especially, the number of clusters is largely controlled by the resolution parameters in the clustering algorithm, and interpretation of the ENS clusters/branches is mostly based on our existing knowledge. Efforts have been made for validation of *in silico* predicted cell types utilizing immunohistochemistry and *in situ* sequencing [15]. These validation experiments are labour intensive and limited by the availability of antibodies. In the next post single-cell era, toolkits for a better integration, unification, interpretation and validation of the accumulated amount of single-cell sequencing data of the ENS are necessary to provide further insights into the ENS and the related disease.

To reduce the sequencing cost and time for the subsequent data analyses, ENS cells are usually enriched by cell sorting before subjected to scRNA-seq. In mice, *Wnt1-Cre* and *Sox10-Cre* reporter mouse lines are frequently used to label the pre-migratory and migratory NC, while *Plp1-GFP* and *Phox2b-GFP* reporters are used to trace enteric glial and neurons, respectively, in later stage of development. As for enriching the enteric neurons, *Baf53b*-Cre pan-neuronal reporter line is the best choice which can mark 95% of HUC/D^+^ neurons in the gut [31]. In general, these reporters can cover 70–95% of the populations of interest, but some rare populations of cells may still not be included for sequencing. Therefore, combined analysis using the datasets generated from different groups with enriched ENS cells using different reporter lines or unsorted gut tissues will help further identification of rare cells. Even though the ENS development is similar in mouse and human and almost all of the genes in mouse share functions with the genes in human, disparate cell types and distinct transcriptome profiles were identified in human and mouse. Thus, the hPSC-based cell- and organoid- models of ENS may represent the alternative tools for functional studies complementary to the mouse models. The scRNA-seq datasets of human ENS cells can be used to assess how hPSC-ENS cells mimic the *in vivo* counterparts. Isolating ENS cells from surrounding tissues is always challenging, especially from human and adult mouse guts. To isolate enteric neurons from human adult colon, Wright et al. micro-dissected out the myenteric plexuses and sequenced 20,167 gut cells, but only 48 neuronal cells were found [32]. May-Zhang et al. used laser-capture microdissection to obtain myenteric ganglia samples from human intestine, the samples could only be used for bulk RNA-seq. To overcome the limitations in profiling rare cell types from a complex tissue, Drokhlyansky et al. overloaded droplets with single nuclei and successfully enriched rare enteric neurons from gut cells. This approach would be useful for large-scale profiling ENS cells from patients [44], but significant technical enhancements are required in order to lower the sequencing cost in the future.

Most of the current single-cell studies focused on building an transcriptional atlas of normal ENS. It will also be crucial to utilize this powerful platform to explore disease-specific gene profiles or cellular drug responses. Measurements of genetic, epigenetic and transcriptomic variations simultaneously at a cell, made possible by single-cell multimodal omics technologies, would enable us to interconnect genetic alternations with gene regulatory network and disease-associated cellular phenotypes. Therefore, more efforts should be put on establishing a standard protocol for tissue processing in order to obtain sufficient amount of single cells of high quality from the patients’ samples for single-cell multimodal omics analyses.

Finally, with accumulation of the multi-stages, multi-species and multimodal single-cell datasets, computational challenges were raised, particularly, for data integration, by which we need to reconcile the heterogeneity of different datasets and tackle with the large amount of missing data inherent in each experiment. For instance, to investigate how progenitor or precursor cells give rise to the more mature neuronal or glial cells at the later stages of development. Morarach et al. identified 12 enteric neuron clusters in the myenteric plexus of the mouse intestine at P21. To address how these neurons were generated, they sequenced the ENS cells at E15.5 and E18.5. By searching for the overlapping mutual neighbours in a PCA space, they aligned cell types at E15.5, E18.5 and P21. Even most of the enteric neurons can be mapped back to the early stages, the fate decisions of some neurons remain obscure [31]. Multi-species integration would also be challenging for the rare populations due to the substantial differences among species. In addition, analysis of transcriptome in single cells may only provide a partial picture of the cell, so a holistic analysis with simultaneous measurements of two or more modalities using single-cell multimodal omics techniques likely allows a more complete understanding of the molecular regulatory circuitries of cells [58]. Multimodal integration of single-cell RNA-seq with scATAC-seq data enables the study of the gene regulatory networks, while integrating single-cell transcriptomic data [16], [43] with their spatial expression data can give us the unprecedented anatomical resolution to physical and molecular interactions between single-cells [18], [36]. These multimodal omics analyses covering multiple layers of information in a cell at a time would further help us to decipher the complex cellular and molecular processes guiding the formation of functional ENS. With an overwhelming amount of sequencing information, the next important step is to properly connect all these single-cell sequencing datasets with biology in order to construct a more holistic map of ENS cells in health and disease.

## 5 Conclusion

Over the past 5 years, a series of single-cell sequencing experiments have been conducted to study the development of ENS, from NC to mature ENS circuit. Numerous stage-specific cell types, markers, fate decision events and regulators directing the development of ENS have been identified and validated. Single-cell multimodal omics analyses of chromatin accessibility, spatial transcriptome and transcriptomics expand our insights into the molecular and cellular regulatory architecture underlying the ENS development. With a rapid evolution of single-cell multimodal omics technologies and the corresponding computational algorithms, we envision that a more detailed cellular and molecular maps of ENS, in both healthy and pathogenic conditions will be established in the near future.

## 6 Grant Support

This work described in this paper was substantially supported by the 10.13039/501100005847HMRF grants (Project no.: 06173306, 08192786) from the Health Department, and the General Research Fund (GRF: HKU 17108019, 17101320) from the Research Grant Council of Hong Kong Special Administrative Region, China Hong Kong.

## CRediT authorship contribution statement

**Zhixin Li:** Writing – original draft. **Elly Sau-Wai Ngan:** Writing – review & editing, Funding acquisition.

## Declaration of Competing Interest

The authors declare that they have no known competing financial interests or personal relationships that could have appeared to influence the work reported in this paper.
